# The Complement System in Dialysis: A Forgotten Story?

**DOI:** 10.3389/fimmu.2018.00071

**Published:** 2018-01-25

**Authors:** Felix Poppelaars, Bernardo Faria, Mariana Gaya da Costa, Casper F. M. Franssen, Willem J. van Son, Stefan P. Berger, Mohamed R. Daha, Marc A. Seelen

**Affiliations:** ^1^Department of Internal Medicine, Division of Nephrology, University Medical Center Groningen, Groningen, Netherlands; ^2^Nephrology and Infectious Diseases Research and Development Group, University of Porto, Porto, Portugal; ^3^Department of Nephrology, Hopsital Braga, Braga, Portugal; ^4^Department of Nephrology, Leiden University Medical Centre, Leiden, Netherlands

**Keywords:** complement, kidney, dialysis, hemodialysis, peritoneal dialysis

## Abstract

Significant advances have lead to a greater understanding of the role of the complement system within nephrology. The success of the first clinically approved complement inhibitor has created renewed appreciation of complement-targeting therapeutics. Several clinical trials are currently underway to evaluate the therapeutic potential of complement inhibition in renal diseases and kidney transplantation. Although, complement has been known to be activated during dialysis for over four decades, this area of research has been neglected in recent years. Despite significant progress in biocompatibility of hemodialysis (HD) membranes and peritoneal dialysis (PD) fluids, complement activation remains an undesired effect and relevant issue. Short-term effects of complement activation include promoting inflammation and coagulation. In addition, long-term complications of dialysis, such as infection, fibrosis and cardiovascular events, are linked to the complement system. These results suggest that interventions targeting the complement system in dialysis could improve biocompatibility, dialysis efficacy, and long-term outcome. Combined with the clinical availability to safely target complement in patients, the question is not if we should inhibit complement in dialysis, but when and how. The purpose of this review is to summarize previous findings and provide a comprehensive overview of the role of the complement system in both HD and PD.

## Introduction

An estimated 2.6 million people are treated for end-stage kidney disease (ESKD) worldwide (1). The majority of ESKD patients are dialysis-dependent. The choice between peritoneal dialysis (PD) and hemodialysis (HD) involves various determinants. Nonetheless, there is no major difference in mortality between HD and PD patients (2). Although considerable progress has been made in survival rates of dialysis patients, cardiovascular morbidity and mortality remain extremely high (3). Both traditional risk factors (such as hypertension, dyslipidemia, and diabetes), as well as non-traditional risk factors (such as oxidative stress, endothelial dysfunction and chronic inflammation), contribute to the high cardiovascular risk (4). In order to lower the high morbidity and mortality rates in dialysis patients, the chronic inflammation seen in these patients must be tackled. The systemic inflammation in dialysis patients can be attributed to the (remaining) uremia, the underlying renal disease, comorbidities, and dialysis-related factors (5). The latter represents an issue that has been present in dialysis throughout history, and still remains unresolved, namely bioincompatibility.

## Biocompatibility

**Figure 1:**
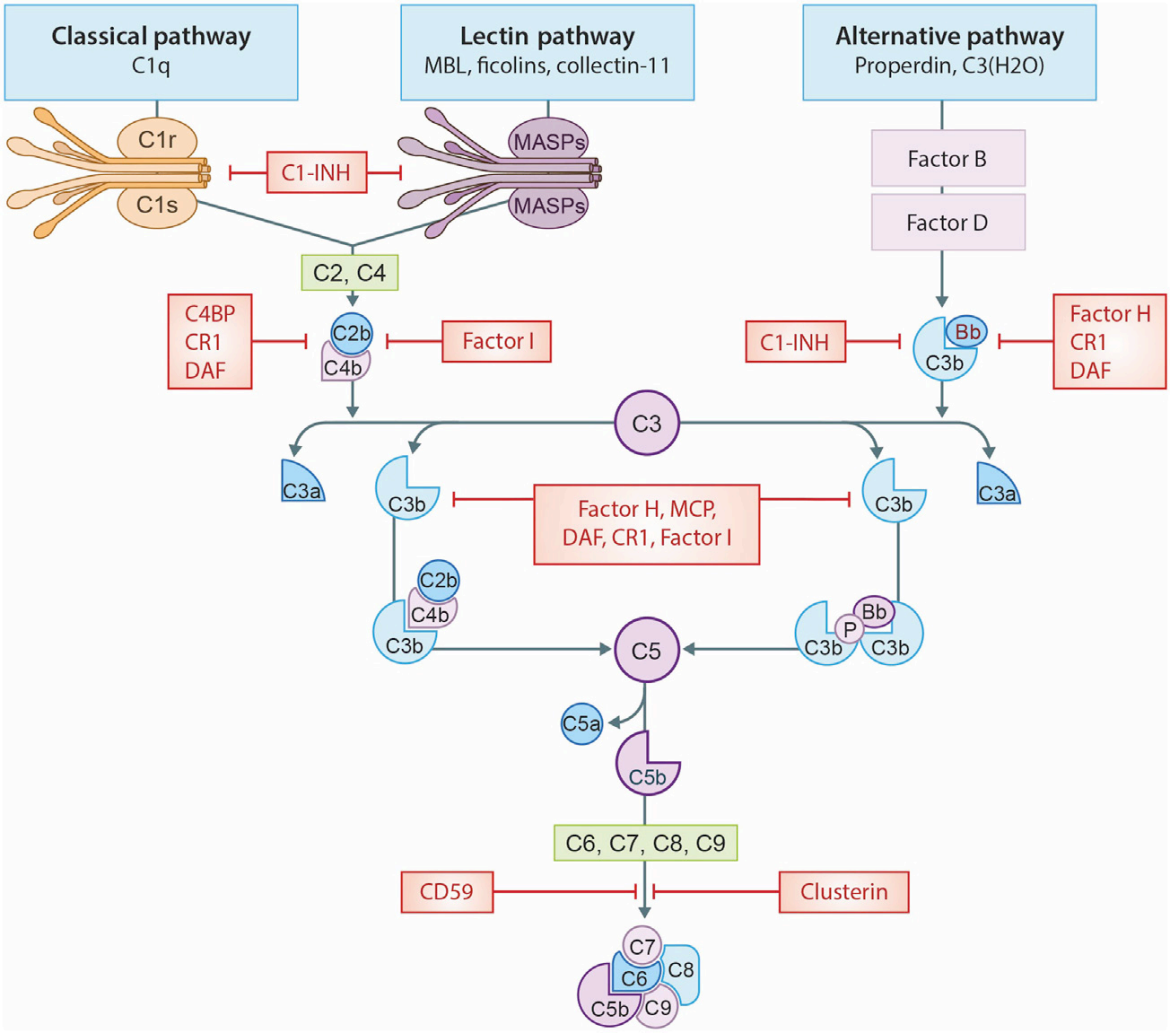
The complement system. A schematic view of activation of the complement system and its regulation. The classical pathway (CP) is initiated by C1q binding to immune complexes or other molecules (e.g., CRP), thereby activating C1r and C1s resulting in the cleavage of C2 and C4 thereby forming the C3-convertase (C4b2b). The lectin pathway (LP) is initiated by mannose-binding lectin (MBL), ficolins, or collectin-11 binding to carbohydrates or other molecules (e.g., IgA), thereby activating MASP-1 and MASP-2, forming the same C3-convertase as the CP. Subsequently, the C3-convertase cleavages C3 into C3a and C3b. Activation of the alternative pathway (AP) occurs *via* properdin binding to certain cell surfaces (e.g., LPS) or by spontaneous hydrolysis of C3 into C3(H_2_O). Next, binding of factor B creates the AP C3-convertase (C3bBb). Increased levels of C3b results in the formation of the C5-convertases, which cleaves C5 in C5a, a powerful anaphylatoxin, and C5b. Next, C5b binds to the surface and interactions with C6–C9, generating the membrane attack complexes (MAC/C5b-9). Several complement regulators (either soluble and membrane-bound) prevent or restrain complement activation. C1 esterase inhibitor (C1-INH) inhibits the activation of early pathway activation of all three pathways, while C4b-binding protein (C4BP) controls activation at the C4 level of the CP and LP. Factor I and factor H regulate the C3 and C5-convertase. Furthermore, the membrane-bound inhibitors include complement receptor 1 (CR1), membrane cofactor protein (MCP) that acts as an co-factors for factor I and decay accelerating factor (DAF) which accelerates the decay of C3-convertases. The membrane-bound regulator Clusterin and CD59 prevents the generation of the C5b-9.

The term “biocompatible” refers to the “capacity of a material/solutions to exist in contact with the human body without causing a (inappropriate) host response” (6). The biocompatibility of the materials used in dialysis remains an important clinical challenge. In HD, the membrane provokes an inflammatory response, as it is the site where blood has direct contact with a foreign surface (7). Additionally, PD fluids containing high glucose levels, hyperosmolarity and acidic pH are considered biologically “unfriendly” and this lack of compatibility causes peritoneal membrane damage (8). Improving biocompatibility in HD and PD is a critical factor to ensure dialysis adequacy and enable long-term treatment (7–9). The challenge of biocompatibility is not confined to dialysis but equally important for other medical devices in contact with either tissue or blood (10). The incompatibility reaction is complex and poorly understood; however, platelets, leukocytes, the complement, and the coagulation system have been shown to be involved (11, 12). In general, incompatibility will lead to inflammation, thrombosis, and fibrosis (11–13). These events will negatively impact the clinical performance and lead to adverse events. The complement system is an important mediator of incompatibility because it can discriminate between self and non-self (14). In accordance, complement has been shown to be activated during cardiopulmonary bypass (15), low-density lipoprotein (LDL) apheresis (16), plasmapheresis (17), and immunoadsorption (18). Additionally, the complement system is also involved in biomaterial-induced complications of medical devices that are not in direct contact with the circulation, such as surgical meshes and prostheses (19, 20). Yet, it should be emphasized that the trigger by which complement is activated is different and depends on the properties of the biomaterial used (20). Proposed mechanisms of indirect complement activation include: (1) immunoglobulin G binding to the biomaterial initiating the classical pathway (CP); (2) lectin pathway (LP) activation by carbohydrate structures or acetylated compounds; or (3) activation of the alternative pathway (AP) by altered surfaces, e.g., plasma protein-coated biomaterials. In addition, complement initiators can also directly bind to the biomaterial, leading to complement activation (20). Irrespective of the pathway, complement activation always leads to the cleavage of C3, forming C3a and C3b (Figure 1). Increased levels of C3b result in the generation of the C5-convertase, cleaving C5 in C5a, a powerful anaphylatoxin and chemoattractant, and C5b. Next, C5b binds to the surface and interacts with C6–C9, forming the membrane attack complex (MAC/C5b-9) (14).

## Hemodialysis

Hemodialysis is a general term including several techniques such as low or high-flux HD (diffusion-based dialysis) and online haemodiafiltration (combined convective and diffusive therapy). Overall, HD remains the most-used form of renal replacement in adult ESKD patients (1). The dialysis membrane can be divided into two main groups, cellulose-based and synthetic membranes (7, 21). In the past, HD membranes were based on cuprophane (a copper-substituted cellulose) because these were inexpensive and thin-walled. The disadvantage of cellulose-based membranes was the immunoreactivity due to the many free hydroxyl-groups. Subsequently, modified cellulosic membranes were developed to improve biocompatibility by replacing the free hydroxyl-groups with different substitutions (especially acetate). The following step was the development of “synthetic” membranes, such as polyacrylonitrile, acrylonitrile-sodium methallyl sulfonate, polysulfone, polycarbonate, polyamide, and polymethylmethacrylate membranes. Nowadays, synthetic membranes are the most commonly used in clinical practice (21). The benefits of these membranes are the varying pore size and reduced immunoreactivity. The complement system is critical in the bioincompatibility of extracorporeal circulation procedures, because complement is abundantly present in blood. Moreover, innate immune activation during HD is a neglected but potentially vital mechanism that contributes to the high morbidity and mortality in these patients (4).

### Complement Activation in HD

In the 1970s, HD was already known to affect the complement system (22). Several studies have since then looked at complement activation during HD, the complement pathway responsible and additional mechanisms contributing to complement activation. In the past, an important adverse event in dialysis was the “first-use syndrome,” named after the fact that these reactions were most severe with new dialyzers. This incompatibility reaction was the result of complement activation by the membrane and closely resembles the pseudo-anaphylactic clinical picture that is nowadays known as complement activation-related pseudoallergy (CARPA) (23, 24). Furthermore, these early studies provided important information on the kinetics of complement activation. During HD, C3 activation, resulting in the generation of C3a, peaks during the first 10–15 min, whereas terminal pathway activation, resulting in C5a and C5b-9 formation occurs at a later stage of dialysis (25). Over the past decades, membranes have been developed with improved biocompatibility. Nonetheless, even with modern “biocompatible” HD membranes significant complement activation still occurs (23, 26, 27). During a single HD session soluble C5b-9 (sC5b-9) levels and C3d/C3-ratios in the plasma increase up to 70% (23, 26). Yet, this is most likely an underestimation of the amount of complement activation, since these values represent fluid phase activation. Complement activation takes place in the plasma (the fluid phase), but also on surfaces (the solid phase) (14). Fittingly, in addition to fluid phase activation, complement depositions have also been shown on the surface of the HD membranes (28).

**Figure 2:**
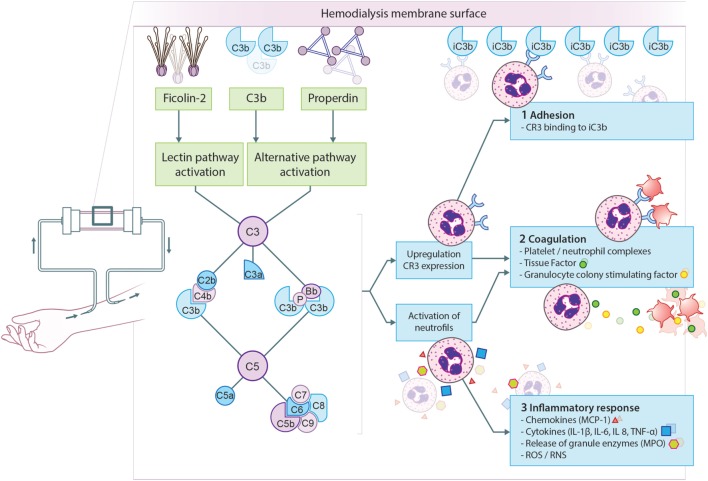
Proposed model for complement activation in hemodialysis (HD). The principal mechanism leading to complement activation in HD is the binding of ficolin-2 to the membrane, resulting in lectin pathway activation. Simultaneously, properdin and/or C3b bind to the membrane resulting in alternative pathway activation. Complement activation will result in the formation of anaphylatoxins (C3a, C5a), opsonins (C3b, iC3b), and the membrane attack complex (C5b-9). First, complement activation leads to the upregulation of complement receptor 3 (CR3) allowing leukocytes to bind C3 fragments deposited on the membrane, leading to leukopenia. Second, CR3 on neutrophils is also important for the formation of platelet-neutrophil complexes, which contributes to thrombotic processes. Furthermore, C5a generation during HD leads to the expression of tissue factor and granulocyte colony-stimulating factor in neutrophils, shifting HD patients to a procoagulant state. Third, complement activation also promotes recruitment and activation of leukocytes resulting in the oxidative burst and the release of pro-inflammatory cytokines and chemokine’s. More specifically, the activation of neutrophils by C5a leads to the release of granule enzymes, e.g., myeloperoxidase (MPO).

Different studies have tried to dissect the pathway responsible for complement activation in HD. Early evidence emerged from a study by Cheung et al., demonstrating AP activation by cellulose membranes (29). Initially, the involvement of the CP or LP was excluded, since it was reported that plasma C4d concentrations remained unaffected during HD (30). However, others were able to show C4 activation by cellulose membranes (31, 32). The increase in C4d levels correlated with the rise in C3d levels, implying that the CP or LP is (at least partly) responsible for the complement activation seen in HD (32). More recently, a role for the LP was demonstrated in complement activation by polysulfone membranes (33, 34). An elegant study by Mares et al., using mass spectrometry, showed a 26-fold change in eluate-to-plasma ratio for ficolin-2 (previously called L-ficolin), suggesting preferential adsorption by the membrane (33). A follow-up study using proteomics analysis of dialyzer eluates revealed that C3c, ficolin-2, mannose-binding lectin (MBL) and properdin were most enriched (28). In addition, plasma ficolin-2 levels decreased by 41% during one HD session, corresponding with the excessive adsorption to the membrane. The decrease in plasma ficolin-2 levels was associated with C5a production and leukopenia during HD (28). The adsorption of properdin to the dialyzer, confirms earlier studies regarding AP activation by HD (28, 29). To summarize, the principal mechanism of complement activation in HD is the binding of MBL and ficolin-2 to the membrane, resulting in LP activation; while, simultaneously, properdin and/or C3b bind to the membrane resulting in AP activation (Figure 2). The latter is supported by the evidence that in C4-deficient patients, systemic complement activation and C3b deposition on the HD membrane are reduced during dialysis but not abolished (31). These results show the importance of the LP, while demonstrating the crucial contribution of the AP.

A second mechanism that could modulate complement activation during HD is the loss of complement inhibitors *via* absorption to the membrane. In HD, polysulfone membranes were shown to absorb factor H and clusterin (28, 33). Factor H is an important inhibitor of C3, while clusterin prevent terminal pathway activation thereby stopping the formation of C5a and C5b-9 (Figure 1) (14). The loss of these inhibitors would cause dysregulation of the AP, leading to further complement activation in the fluid phase (i.e., in the circulation) in HD patients.

### Effector Functions and Clinical Implications of Complement Activation

Complement activation will lead to the generation of effector molecules, which can result in a variety of biological responses (14). In HD, the most important effector functions of complement activation are the induction of inflammation, promoting coagulation and impaired host defense due to accelerated consumption of complement proteins (20, 35, 36).

The generation of C3a and C5a during HD promotes recruitment and activation of leukocytes (37, 38). Leukocyte activation results in the oxidative burst and the release of pro-inflammatory cytokines and chemokine’s, such as interleukin (IL)-1β, IL-6, IL-8, tumor necrosis factor-α, monocyte chemoattractant protein-1, and interferon-γ. More specifically, the activation of PMNs by C5a leads to the release of granule enzymes such as myeloperoxidase and elastase (39–41). Furthermore, complement activation in HD patients results in the upregulation of adhesion molecules on leukocytes, especially complement receptor 3 (CR3). The C5a-activated leukocytes will then bind C3 fragments (iC3b) deposited on the membrane *via* CR3, leading to leukopenia (20, 28, 39). Likewise, CR3 on PMNs is also important for the formation of platelet–PMN complexes, which can contribute to both inflammatory and thrombotic processes (42). The crosstalk between activation of the complement and coagulation system has correspondingly been described in HD. It has been demonstrated that C5a generation during HD leads to the expression of tissue factor and granulocyte colony-stimulating factor in PMNs, shifting HD patients to a procoagulative state (35). In conformity, plasma C3 levels have been shown to positively correlated with a denser clot structure in HD patients (43). On the other hand, the coagulation system has also been shown to impact complement activation (44).

Inflammation and coagulation are principally involved in the pathogenesis of cardiovascular disease. Accordingly, complement has been associated to the susceptibility to cardiovascular disease in HD patients (26, 27, 45–47). Plasma C3 levels, prior to a HD session, were found to be higher in patients who develop a cardiovascular event (CV-event) than HD patients who remained event-free. Moreover, an association was found between C3 levels and the development of CV-events (27). A similar trend of higher C3 levels in HD patients who develop a CV-event was seen in our study (26). A possible explanation would be that higher C3 levels prior to HD might reflect the potential for HD-evoked complement activation. Additionally, another association was found for baseline sC5b-9 levels with the occurrence of CV-events as well as mortality. This association was complex and showed an U-shaped relationship, indicating that both high and low sC5b-9 levels led to a higher risk, whereas HD patients with mid-range values were protected (27). Furthermore, a common factor H gene polymorphism was found to be an independent predictor of cardiovascular disease in HD patients (47). Homozygous HD patients for the Y402H polymorphism had an odds ratio of 7.28 for the development of CV-events compared to controls. This polymorphism affects the binding sites for heparin and C-reactive protein (CRP) and it has, therefore, been hypothesized that the reduced binding of factor H to the patient’s endothelial cells would increase their risk of a CV-event. Alternatively, the link between the factor H polymorphism and the cardiovascular risk in HD patients could be mediated through CRP, since factor H binds CRP and thereby undermines its pro-inflammatory activity (48, 49). The Y402H polymorphism of factor H results in inadequate binding to CRP and thus leaves the pro-inflammatory activity of CRP unchecked. Furthermore, several studies have demonstrated that CRP levels in HD patients are associated to cardiovascular mortality (50–52). *Buraczynska* et al. revealed that in HD patients the complement receptor 1 (CR1) gene polymorphism C5507G is independently associated with the susceptibility for cardiovascular disease (46). Whether this effect is mediated *via* the complement inhibitory capacity of CR1 or *via* the recently discovered function of CR1 in the binding and clearance of native LDL remains to be elucidated (53). Another study showed that low serum C1q-adiponectin/C1q ratios were linked to cardiovascular disease in HD patients (45). The mechanism behind this connection is not understood but it has been demonstrated that adiponectin protects against activation of C1q-induced inflammation (54). Thus, in HD patients increased complement activation, as well as increased complement activity and the loss of complement inhibitors have all been linked to a higher risk of cardiovascular disease (Table 1). Recently, our group showed that low MBL levels are also associated with the occurrence of cardiovascular disease in HD patients (26). The higher risk in these patients was attributed to CV-events linked to atherosclerosis. In support of this, low MBL levels have been linked to enhanced arterial stiffness in HD patients (55). Accordingly, Satomura et al. demonstrated that low MBL levels were an independent predictor of all-cause mortality in HD patients (56). We, therefore, postulate that in HD patients, low MBL levels promote cardiovascular disease by enhancing atherosclerosis due to the inadequate removal of atherogenic particles.

**Table 1:** The association between complement proteins and morbidity and mortality in HD patients.

Study	Complement protein	Outcome	Association^a^	Possible mechanism
Poppelaars et al. (26, 67)	MBL levels	CV-events	Low MBL levels OR = 3.98 (1.88–8.24)	Low MBL levels promote atherosclerosis due to the inadequate removal of atherogenic particles
Satomura et al. (56)	MBL levels	All-cause mortality	Low MBL levels OR = 7.63 (2.24–25.96)	Low MBL levels promote atherosclerosis due to the inadequate removal of atherogenic particles
Kishida et al. (45)	C1q-adiponectin levels	CV-events	Low C1q-adiponectin levels	Adiponectin protects against activation of C1q-induced inflammation
Lines et al. (27)	C3 levels	CV-events	Higher C3 levels (per 0.1 mg/ml) HR = 1.20 (1.01–1.42)	Increased complement activity
Lines et al. (27)	sC5b-9 levels	CV-events	Low and high sC5b-9 levels	(1) Increased complement activation. (2) Complement depletion by local complement activation on the HD membrane
			U-shaped relationship	
		All-cause mortality	Low and high sC5b-9 levels	
			U-shaped relationship	
Buraczynska et al. (47)	Factor H gene polymorphism (Y402H)	CV-events	The CC genotype OR = 7.28 (5.32–9.95)	(1) The loss of complement inhibition, leading to complement activation. (2) Reduced binding of factor H to endothelial cells.
Buraczynska et al. (46)	CR1 gene polymorphism (C5507G)	CV-events	The GG genotype OR = 3.44 (2.23–5.3)	(1) The loss of complement inhibition, leading to complement activation. (2) Reduced binding and clearance of native low-density lipoprotein by CR1.

*^a^Data are presented as hazard or OR plus 95% confidence interval*.

*OR, odds ratio; HR, hazard ratio; HD, hemodialysis; MBL, mannose-binding lectin; CR1, complement receptor 1; CV-event, cardiovascular event; sC5b-9, soluble C5b-9*.

In HD patients, little is known about the changes in complement components overtime. The plasma levels of C3 have been shown to decrease after 12 months compared to baseline (27). In this study, the C3 levels also negatively correlated with the dialysis vintage. In addition, the ability to activate complement has also been shown to be decreased in HD patients compared to healthy controls (23). In theory, these acquired deficiencies of complement proteins could explain the higher infection and sepsis risk seen in HD patients. Conversely, there was no association between low MBL levels and the risk of infection in HD patients (57). However, the authors concluded that this might be due to a compensation mechanism of higher ficolin-2 and MASP-2 levels in MBL-deficient individuals. Furthermore, another study found that long-term HD patients have decreased levels of clusterin, factor B and factor H compared to short-term HD patients (58). Thus far, no study has analyzed the link between HD-acquired complement deficiencies and infection risk. The clinical consequences of the HD-induced ficolin-2 reduction would be the most interesting to examine (28, 33). It is highly likely that this reduction would have a tremendous impact on HD patients’ health and outcome. A genetic deficiency in ficolin-2 has not been reported to date, highlighting the essential function of this component within host defense. In conformity, ficolin-2 has been shown to be involved in the elimination of numerous pathogens (59).

### Therapeutic Options

Several types of interventions have been proposed or tested in HD patients to decrease inflammation or target cardiovascular risk factors with mixed success. Hence, the clinical need for better therapeutic options that limit the inflammation and decrease cardiovascular risk in HD patients is on-going. The complement system is considered to be a promising target during HD to limit the inflammation and decrease cardiovascular risk (60). Therapies modulating HD-induced complement activation have focused on three treatment strategies: (1) reduction in the complement activating-capacity of the HD membrane; (2) the use of non-specific complement inhibitors (e.g., anticoagulants with a complement inhibitory property); and (3) specific complement-directed therapies.

Prevention is better than cure; therefore, creating a truly biocompatible membrane would, therefore, be ideal to prevent complement activation during HD. Much progress has been made with the development of more biologically compatible membranes by surface modifications and reducing protein retention. Today, the most common HD membranes contain sulfonyl-groups (7). To further improve biocompatibility, it is vital to understand the structures that initiate complement activation as it has the potential to develop HD membranes with enhanced biocompatibility. In modern HD membranes, ficolin-2 seems to be an important mediator in HD-induced complement activation (28, 33). Ficolin-2 is unfortunately a highly promiscuous molecule with numerous binding partners, several of which are acetylated compounds (59).

Anticoagulants have been used extensively to render biomaterial-blood incompatibility, through inhibition of the coagulation, contact and complement system. The effect of citrate anticoagulation on complement activation has widely been studied in HD. Citrate has calcium-chelating properties and thereby reduces complement activation (61, 62). During the initial phase of HD with cellulose membranes, citrate anticoagulation reduced C3a levels by almost 50% compared to heparin (63). However, no complement inhibition was seen by citrate anticoagulation during HD in other studies with cellulose or synthetic membranes (64–66). Heparinoids are also known to prevent complement activation, although this inhibition is strictly concentration dependent (67). Although heparin has been tested extensively in HD, sadly none of these studies determined the effect on complement activation.

In the past decade, numerous complement inhibitors have been developed; two are currently used in the clinics and others are now undergoing clinical trials. Purified C1 esterase inhibitor (C1-INH) is a protease that is clinically used to treat hereditary angioedema. Eculizumab, a C5 antibody is used for the treatment of paroxysmal nocturnal hemoglobinuria and atypical hemolytic uremic syndrome (14, 68). In HD, specific complement-directed therapies have predominantly been evaluated in experimental settings, still valuable information has been uncovered and shown that the use of complement inhibitors are a promising tool to reduce the inflammatory response and subsequent consequences in these patients (60). The potential of complement inhibition in HD is further underlined by the successful use of complement inhibitors for biomaterial-induced complement activation in cardiopulmonary bypass systems (19). In patients undergoing cardiopulmonary bypass surgery, treatment with soluble CR1 (sCR1/TP30), an inhibitor of C3, lead to a decrease in mortality and morbidity as well as a reduced need for intra-aortic balloon pump support (69). Consequently, soluble complement inhibitors may be equally effective in HD, since there is the recurrent need of complement inhibition for short periods. Specifically, the short half-life of sCR1 matches the need for restricted complement inhibition in HD, which is only needed during dialysis, after which complement activity should be reestablished between sessions. This approach would also prevent complications of long-term immunosuppression. In a pre-clinical monkey model of HD, another C3-inhibitor (compstatin) was used to attenuate HD-induced complement activation (70). Despite the use of HD membranes with high biocompatibility and standard heparin treatment in their study, severe complement activation still occurred in monkeys. In this study, animals received a bolus injection prior to the HD and a continuous infusion of compstatin during the 4 h HD procedure. Treatment completely blocked complement activation and C3 activation products stayed at basal levels throughout the HD session. Strikingly, a second treatment regimen with only a bolus injection of compstatin at the start of the session was also sufficient to abolished complement activation throughout the procedure. Furthermore, complement inhibition lead to the increase of IL-10, an anti-inflammatory cytokine. Unfortunately, the effect of complement inhibition on other inflammatory markers could not be assessed, since one HD session was insufficient to induce substantial levels of pro-inflammatory cytokines. Next to inhibition of the central component C3, blockage of early complement components may be equally successful. C1-INH forms a therapeutic option, since HD leads to LP activation and C1-INH could attenuate this (67). Additionally, C1-INH also affects the coagulation and contact system, which could add to the success of this therapeutic approach. Given the strong involvement of complement activation effector molecules in HD, more specifically C5a, another attractive option would be the inhibition of C5 or C5a-receptor antagonists (C5aRA) (35). This could be either done by the anti-C5 antibody or by C5aRA. Eculizmab blocks the generation of C5a and C5b-9 and could thus be more effective than C5aRA. However, the long half-life and the high costs form important disadvantages. In contrast, C5aRA tends to be more cost-effective (71). These drugs could significantly reduce activation of leukocytes and thereby inflammation in HD. Currently, the most likely candidate to be used in HD is PMX-53, a C5aRA, since this compound is currently tested in different clinical trials (72). Another promising approach is coating biomaterials with complement inhibitors (20). One of these molecules, the 5C6 peptide is a molecule that has strong binding affinity toward factor H without modifying its inhibitory activity. More importantly, polystyrene surfaces coated with 5C6 were shown to bind factor H and thereby prevent complement activation when exposed to human plasma, thus enhancing biocompatibility (73). However, it is unknown whether the reduction of systemic factor H levels by 5C6 during HD could have undesirable consequences, such as seen in factor H-deficient individuals. Finally, the cost of the different complement inhibitors should be taken into account, considering the high frequency of treatments required in HD patients.

## Peritoneal Dialysis

Peritoneal dialysis is the most common used dialysis technique at home and is equally effective as HD for the treatment of CKD (74). Nevertheless, the advantages of PD include; better preservation of residual renal function, lower infectious risk and higher satisfaction rates. Despite the good results seen with PD, this dialysis technique remains underused (1). In PD, unlike in HD, no synthetic membrane is used. In contrast, the peritoneum in the abdominal cavity of the patients acts as a semi-permeable membrane allowing diffusion between the dialysis fluid and the circulation. The osmotic gradient during PD is based on high glucose levels in the dialysate. However, glucose acts as a double edge sword, since it serves as an osmotic agent but it is also responsible for the incompatibility reaction. The peritoneal membrane is made up of an inner mesothelial layer and these cells are, therefore, directly in contact with the dialysis fluid. Long-term exposure to dialysate leads to tissue remodeling of this layer resulting in peritoneal fibrosis (75). This progressive fibrosis forms a major limitation for chronic PD treatment. Another common complication in PD is peritonitis (76). Patients who develop peritonitis can have irreversible peritoneum damage, PD failure and significant morbidity or even mortality. For this reason, avoiding PD failure due to peritonitis or fibrosis remains a challenge for nephrologists (77).

### Complement Activation in PD

The link between the complement system and PD seems less obvious, because there is no direct contact with blood. However, mesothelial cells produce and secrete different complement factors, including C4, C3, and C5 till C9 (78, 79). In accordance, different studies have found the presence of complement in the peritoneal dialysate. Additionally, the amount of C3 in the PD fluid does not depend on the serum concentration, suggesting that the C3 originates from local production (80). The study by Oliveira et al. found strong protein abundance of Factor D in six adult PD patients (81), whereas a similar approach in 76 PD patients by Wen et al. found significant protein expression of C4 and C3 only (82). Altogether, proteomic analyses of the dialysate of healthy PD patients has revealed the presence of C4, C3, Factor B, Factor D, Factor H, Factor I, and C9 (81–85). Proteomic profiling in the peritoneal fluid of children identified a total number of 189 proteins, of which 18 complement components (84). The discrepancies between the various proteomic studies could be explained by differences in the underlying cause of renal failure, since diabetic patients on PD have been shown to have lower levels of C4 in the dialysate compared to controls (83). Obviously, other patient’s characteristics such as ethnicity and differences in the accuracy and sensitive of the analysis have to be taken into account as well. Complement production by mesothelial cells has been shown to be increased in uremic patients and it can be further stimulated upon exposure to PD solutions containing glucose (78, 79). Next to complement production; mesothelial cells also express important complement regulators; e.g., MCP, DAF, and CD59 (79, 80).

Systemically, PD patients have lower MBL levels compared to HD patients and healthy controls, even after adjusting for the effect of mutations (86). This could indicate loss of systemic MBL *via* the peritoneal route, independent of the reduced renal function. However, MBL has so far not been assessed in peritoneal dialysates. Furthermore, serum levels of C1q, C4, C3d, factor D, and properdin were shown to be higher in pediatric PD patients compared to healthy controls, however, not in comparison to patients with ESKD (87). Overall, the higher plasma levels of the complement components are likely caused by increased synthesis by the liver due to the pro-inflammatory state in ESKD patients. Moreover, the increased levels of C3d in PD patients are believed to be the consequence of reduced elimination of factor D by the kidney, creating enhanced AP activation. However, while systemic complement activation (the fluid phase) is similar between PD patients and patients with ESKD, higher intravascular complement depositions (solid phase) have been shown in children with PD compared to non-PD children with ESKD. Omental and parietal arterioles from PD patients demonstrated a higher presence of C1q, C3d, and C5b-9 (88).

Evidence has also been provided for complement activation in the peritoneal cavity in PD patients (80, 89). Previously, it was demonstrated that the dialysate/serum ratios of factor D and C3d were elevated in PD, whereas the dialysate/serum ratios of C3, C4, and properdin were decreased (89). The high dialysate levels of C3d demonstrate local complement activation, while the comparatively low dialysate/serum ratios of complement components are likely caused by intraperitoneal complement consumption. In accordance, the presence of sC5b-9 in the peritoneal dialysate has also been shown. In the dialysate of PD patients, sC5b-9 levels up to 200 pg/μg of total protein level have been reported (80). Considering the high molecular weight of sC5b-9 (>1,000 kDa), it is very likely that the sC5b-9 in the dialysate is produced in the peritoneal cavity and does not originate from the circulation.

**Figure 3:**
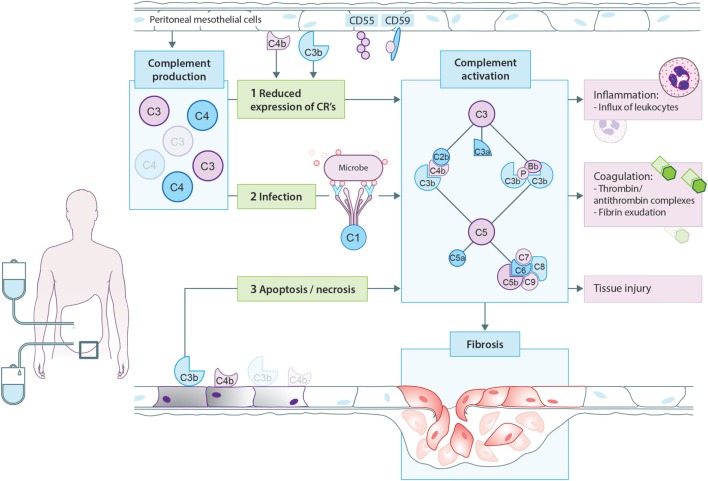
Proposed model for complement activation in peritoneal dialysis (PD). In PD patients, mesothelial cells produce and secrete different complement factors. One of the proposed mechanisms of complement activation in PD patients is that PD therapy decreases the expression of complement regulators such as CD55 and CD59 on the peritoneal mesothelium, leading to local complement activation. In addition, cellular debris as a result of direct peritoneal damage by bioincompatible PD fluids as well as antibodies against microorganisms could contribute to local complement activation during PD. Complement activation will result in the formation of anaphylatoxins (C3a, C5a), opsonins (C3b, iC3b), and the membrane attack complex (C5b-9). First, complement activation leads to the influx of leukocytes, predominantly neutrophils. Second, complement activation increased the production of thrombin anti-thrombin complexes and fibrin exudation on the surface of the injured peritoneum. Altogether, these events indicate the activation of the coagulation system. Third, complement activation during PD leads to direct damage of the peritoneum. Moreover, recent evidence suggests that complement activation promotes the progression to fibrosis after tissue injury. In PD, complement activation could stimulate mesothelial cells to undergo epithelial-to-mesenchymal transition, resulting in the accumulation of myofibroblasts and consequently peritoneal fibrosis.

One of the proposed mechanisms of complement activation in PD patients is that PD therapy modifies the expression of complement regulators on the peritoneal mesothelium, leading to local complement activation (Figure 3). In accordance, CD55 expression is lower on mesothelial cells from PD patients than non-CKD patients and the reduced expression of CD55 is accompanied by higher peritoneal levels of sC5b-9 (80). Likewise, complement regulators were also shown to be downregulated in arterioles of PD patients. Furthermore, the C5b-9 deposition seen in the arterioles of PD patients correlated with the level of dialytic glucose exposure (88). However, this is probably not the only mechanism responsible for complement activation in PD patients. Hypothetically, cellular debris as a result of direct peritoneal damage by bioincompatible PD fluids as well as antibodies against microorganisms could contribute to local complement activation during PD. Unfortunately, most of the reviewed studies are relatively old and there is, therefore, a need for novel studies to assess the effect of newer PD solutions on complement production and activation.

### Effector Functions and Clinical Implications of Complement Activation

During PD, complement activation occurs locally within the peritoneal cavity and leads to the generation of opsonins, anaphylatoxins, and the MAC. The effects of complement activation during PD include the induction of tissue injury, inflammation, coagulation, and fibrosis. However, complement activation in PD patients has also been linked to long-term effects such as cardiovascular risk (88). In different experimental models, complement activation during PD leads to direct damage of the peritoneum. The complement-induced peritoneal damage seems to be mediated *via* activation of the terminal pathway, specifically C5a and C5b-9 (90–92). Additionally, complement activation leads to inflammation. In a rat model of peritoneal fluid infusion, the numbers of neutrophils increased significantly overtime, and this process was largely dependent on C5 activation. In conformity, intraperitoneal injections with C3a and C5a in mice lead to the influx of leukocytes, predominantly neutrophils (93). The effect of C5a is mediated *via* C5aR1, while the effect of C3a is presumably mediated *via* the C3a-receptor. The crosstalk between activation of the complement and coagulation system has also been described in PD. Thrombin anti-thrombin complexes increased significantly in experimental models of PD and this process was partly dependent on C5 activation (92). Mizuno et al. showed that intraperitoneal complement activation leads to fibrin exudation on the surface of the injured peritoneum (94). Altogether these findings indicate that activation of the coagulation system by the PD therapy is at least (partly) complement dependent. The fibrin exudate can also be a sign of PD-associated fibrosis.

The link between fibrosis and complement is relatively new; nevertheless, recent evidence suggests that complement activation promotes the progression to fibrosis after tissue injury (95). In PD, high peritoneal transport is associated with progression of peritoneal fibrosis (96). Proteomics analysis of PD fluid showed enhanced expression of C3 in patients with high transporter status, while expression of C4 is lower in low transporters (82, 97). Furthermore, in PD mesothelial cells undergo epithelial-to-mesenchymal transition, resulting in the accumulation of myofibroblasts and consequently peritoneal fibrosis (98). In other disease models, complement has been shown to induce epithelial-to-mesenchymal transition (99). This effect is mediated *via* the C5aR1, since in rodent models of infection–induced peritoneal fibrosis C5aR1^−/−^ mice were protected against fibrosis (100). The C5aR1 is also involved in the production of profibrotic and inflammatory mediators by peritoneal leukocytes (100). In addition, Bartosova et al. reported that in the peritoneal arterioles of PD patient’s, high abundance of complement deposition was found to correlate with TGF-b signaling (88). More specifically, C1q and C5b-9 deposition were associated with an increased phosphorylation of SMAD2/3, and enhanced vasculopathy. Interestingly, the TGF-b–SMAD pathway has also been recently linked to cardiovascular disease (101). Encapsulating peritoneal sclerosis is another long-term complication of PD, which is the result of abnormal thickening and fibrosis of the peritoneum, leading to a fibrous cocoon thereby encapsulating the intestines causing obstruction (102). The exact cause of this rare complication is unknown, but it is linked to the bioincompatibility of the glucose-based PD solutions (103). The bioincompatibility of these solutions presumably promotes the expression TGF-b, thereby stimulating the transition of mesothelial cells to myofibroblasts. Recently, a prospective proteomics study identified complement components as a possible biomarker of encapsulating peritoneal sclerosis (85). Factors B and factor I were elevated in the PD fluid of patients up to 5 years prior to developing encapsulating peritoneal sclerosis. In patients with stable membrane function, factor I was present in the PD fluid in lower amounts and decreased overtime, while factor B was barely detectable in the PD fluid of controls. However, whether the elevated levels of these complement factors are merely an acute phase response or involved in the pathogenesis remains to be investigated. Yet, based on the current literature, complement activation is likely to play a role in the mechanisms of peritoneal fibrosis. Nevertheless, additional studies are needed to further elucidate the specific role of the complement system in this process.

Peritonitis is another common complication with significant morbidity and mortality. Complement has been proposed to be involved in the risk of PD patients for peritonitis. First, a variation in the FCN2 gene was shown to be more prevalent in PD patients with a history of peritonitis (104). In addition, local activation will lead to a further decline of already low levels of complement components in PD fluid and may thereby additionally impair host defense. Complement activation products have also been suggested as a biomarker during peritonitis. Mizuno et al. showed that C4, C3, and sC5b-9 levels in the peritoneal fluid are significantly higher in PD patients with poor prognosis after peritonitis (105). Complement markers in peritoneal fluid have, therefore, the potential to serve as a biomarker for the prediction of the prognosis of PD-related peritonitis. Finally, the risk of peritonitis could form a major Achilles heel for complement inhibition in PD.

### Therapeutic Options

Treatment aimed at attenuating or blocking complement activation in PD has mostly focused on the terminal pathway. The advantage of this approach is the elimination effector functions of C5a and/or C5b-9, while proximal complement functions stay intact. *In vitro*, inhibition of the C5aR1 on peritoneal leukocytes, isolated from PD fluid, reduced bacteria-induced profibrotic (TGF-β) and inflammatory (IL-6 and IL-8) mediator production (100). In addition, the systemic administration of a C5aR1 antagonist in a rat model of PD prevented influx of inflammatory cells and reduced tissue damage of the peritoneal cavity (91). Furthermore, blockage of C5 in PD improved ultrafiltration and additionally reduced activation of the blood clotting system (92). Other studies have confirmed these results; showing that C5 blockade significantly increased the ultrafiltration volume *via* reduced peritoneal glucose transport, most likely by preventing C5a-induced vasodilatation (106). In contrast, C3 inhibition through complement depletion by cobra venom factor, also led to diminished chemoattractant release, neutrophil recruitment and enhanced ultrafiltration (106). Anticoagulants have also been tested for the treatment of the inflammatory reaction to PD fluids (106, 107). The addition of low-molecular-weight heparin to the PD fluid not only prevented thrombin formation but also inhibited the complement activation, neutrophil recruitment, and improved ultrafiltration (107). In brief, results about complement inhibition in PD look promising, but many hurdles remain to be solved.

## Conclusion

In conclusion, biocompatibility remains an important clinical challenge within dialysis. Due to bioincompatibility, complement is systemically activated during HD, while PD leads to local complement activation. Moreover, important effector functions of complement activation include promoting inflammation and coagulation. In addition, long-term complications of dialysis, such as infection, fibrosis, and cardiovascular events, are linked to the complement system. These results indicate the possibility for complement interventions in dialysis to improve biocompatibility, dialysis efficacy, and long-term outcome.

## Author Contributions

FP and MG performed the literature search. MD, SB, and MS helped with the interpretation of the literature. BF and CF provided the review with clinical information and the clinical relevance. FP, BF, and MG wrote the review. WS, CF, SB, MD, and MS critically reviewed the manuscript prior to submission.

## Conflict of Interest Statement

The authors declare that the research was conducted in the absence of any commercial or financial relationships that could be construed as a potential conflict of interest.

## Abbreviations

AP, alternative pathway; C1-INH, C1 esterase inhibitor; C3aR, C3a-receptor; C5aR, C5a-receptor; C5aRA, C5a-receptor antagonist; CARPA, complement activation-related pseudo allergy; MCP, membrane cofactor protein; CD55, decay accelerating factor; CD59, membrane attack complex-inhibitory protein; CP, classical pathway; CR1, complement receptor 1; CR3, complement receptor 3; CRP, C-reactive protein; CV-event, cardiovascular event; DAF, decay accelerating factor; ESKD, end-stage kidney disease; HD, hemodialysis; IgG, immunoglobulin G; IgM, immunoglobulin M; IL, interleukin; LDL, low-density lipoprotein; LP, lectin pathway; MAC, membrane attack complex; MBL, mannose-binding lectin; MCP-1, monocyte chemoattractant protein-1; PD, peritoneal dialysis; sC5b-9, soluble C5b-9; sCR1, soluble complement receptor 1; TGF-β, tumor growth factor beta; TNF-α, tumor necrosis factor alfa.
